# Single-shot 3D coherent diffractive imaging of core-shell nanoparticles with elemental specificity

**DOI:** 10.1038/s41598-018-26182-1

**Published:** 2018-05-29

**Authors:** Alan Pryor, Arjun Rana, Rui Xu, Jose A. Rodriguez, Yongsoo Yang, Marcus Gallagher-Jones, Huaidong Jiang, Krishan Kanhaiya, Michael Nathanson, Jaehyun Park, Sunam Kim, Sangsoo Kim, Daewoong Nam, Yu Yue, Jiadong Fan, Zhibin Sun, Bosheng Zhang, Dennis F. Gardner, Carlos Sato Baraldi Dias, Yasumasa Joti, Takaki Hatsui, Takashi Kameshima, Yuichi Inubushi, Kensuke Tono, Jim Yang Lee, Makina Yabashi, Changyong Song, Tetsuya Ishikawa, Henry C. Kapteyn, Margaret M. Murnane, Hendrik Heinz, Jianwei Miao

**Affiliations:** 1 Department of Physics & Astronomy and California NanoSystems Institute, University of California, Los Angeles, CA 90095 USA; 2 Department of Biological Chemistry, UCLA-DOE Institute for Genomics and Proteomics, University of California, Los Angeles, CA 90095 USA; 3 School of Physical Science and Technology, ShanghaiTech University, Shanghai, 201210 China; 4 Department of Chemical and Biological Engineering, University of Colorado at Boulder, Boulder, CO 80309 USA; 5 RIKEN SPring-8 Center, Kouto 1-1-1, Sayo, Hyogo, 679-5148 Japan; 6 Pohang Accelerator Laboratory, Pohang, 790-784 South Korea; 7 Department of Physics, Pohang University of Science and Technology, Pohang, 790-784 South Korea; 8 Department of Chemical and Biomolecular Engineering, National University of Singapore, 10 Kent Ridge Crescent, Singapore, 119260 Singapore; 9 Department of Physics and JILA, University of Colorado and National Institute of Standards and Technology, Boulder, CO 80309 USA; 10 Japan Synchrotron Radiation Research Institute, Kouto 1-1-1, Sayo, Hyogo, 679-5198 Japan

## Abstract

We report 3D coherent diffractive imaging (CDI) of Au/Pd core-shell nanoparticles with 6.1 nm spatial resolution with elemental specificity. We measured single-shot diffraction patterns of the nanoparticles using intense x-ray free electron laser pulses. By exploiting the curvature of the Ewald sphere and the symmetry of the nanoparticle, we reconstructed the 3D electron density of 34 core-shell structures from these diffraction patterns. To extract 3D structural information beyond the diffraction signal, we implemented a super-resolution technique by taking advantage of CDI’s quantitative reconstruction capabilities. We used high-resolution model fitting to determine the Au core size and the Pd shell thickness to be 65.0 ± 1.0 nm and 4.0 ± 0.5 nm, respectively. We also identified the 3D elemental distribution inside the nanoparticles with an accuracy of 3%. To further examine the model fitting procedure, we simulated noisy diffraction patterns from a Au/Pd core-shell model and a solid Au model and confirmed the validity of the method. We anticipate this super-resolution CDI method can be generally used for quantitative 3D imaging of symmetrical nanostructures with elemental specificity.

## Introduction

Core-shell nanoparticles exhibit unique electronic, chemical, catalytic and optical properties that have found applications across many disciplines^1–4^. Conventional methods to characterize these nanoparticles rely on electron microscopy, scanning probe microscopy, x-ray diffraction, scattering and spectroscopic techniques^1–4^. Although atomic electron tomography (AET) has recently been developed to determine the 3D structure of nanoparticles at the single atomic level, AET requires that the sample be thin enough to mitigate the dynamical scattering effect^5–7,8^. Scanning probe microscopy is limited to studies of surface structures, while x-ray diffraction and scattering methods only provide average structural information^1,3,4^. In contrast, CDI can be used to determine the 3D internal electron density of thick samples at high resolution^9,10^. Following the first experimental demonstration in 1999^11^, a number of CDI methods have been developed and applied to image a broad range of samples in physics, chemistry, materials science, nanoscience and biology^9,10,12–34^.

With the advent of x-ray free electron lasers (XFELs) that produce extremely intense and short x-ray pulses^9,35,36^, CDI has opened the door for high-resolution imaging of both physical and biological specimens based on the diffraction-before-destruction scheme^37,38^. However, the XFEL pulse is very intense and destroys the specimen after one exposure. Therefore, it is desirable to find a way to obtain 3D structure information from a single x-ray pulse. One method to achieve 3D structural determination from a single sample orientation is to use the curvature of the Ewald sphere together with additional constraints such as symmetry and sparsity^39–44^. Note that symmetry has been widely applied to image 3D virus structures using cryo-electron microscopy^45^. Here, we implemented a super-resolution CDI technique to extract 3D structural information of core-shell nanoparticles beyond the diffraction signal. We reconstructed the 3D electron density of individual Au/Pd core-shell nanoparticles from single-shot diffraction patterns with 6.1 nm resolution. By exploiting CDI’s ability for quantitative reconstructions, we applied high-resolution model fitting to determine the Au core size and the Pd shell thickness to be 65.0 ± 1.0 nm and 4.0 ± 0.5 nm, respectively. We quantified the 3D elemental distribution inside the nanoparticle with an accuracy of 3%. We further validated the technique using simulated diffraction patterns with noise and missing data.

## Results

### XFEL experiment and 3D reconstruction of core-shell nanoparticles

**Figure 1:**
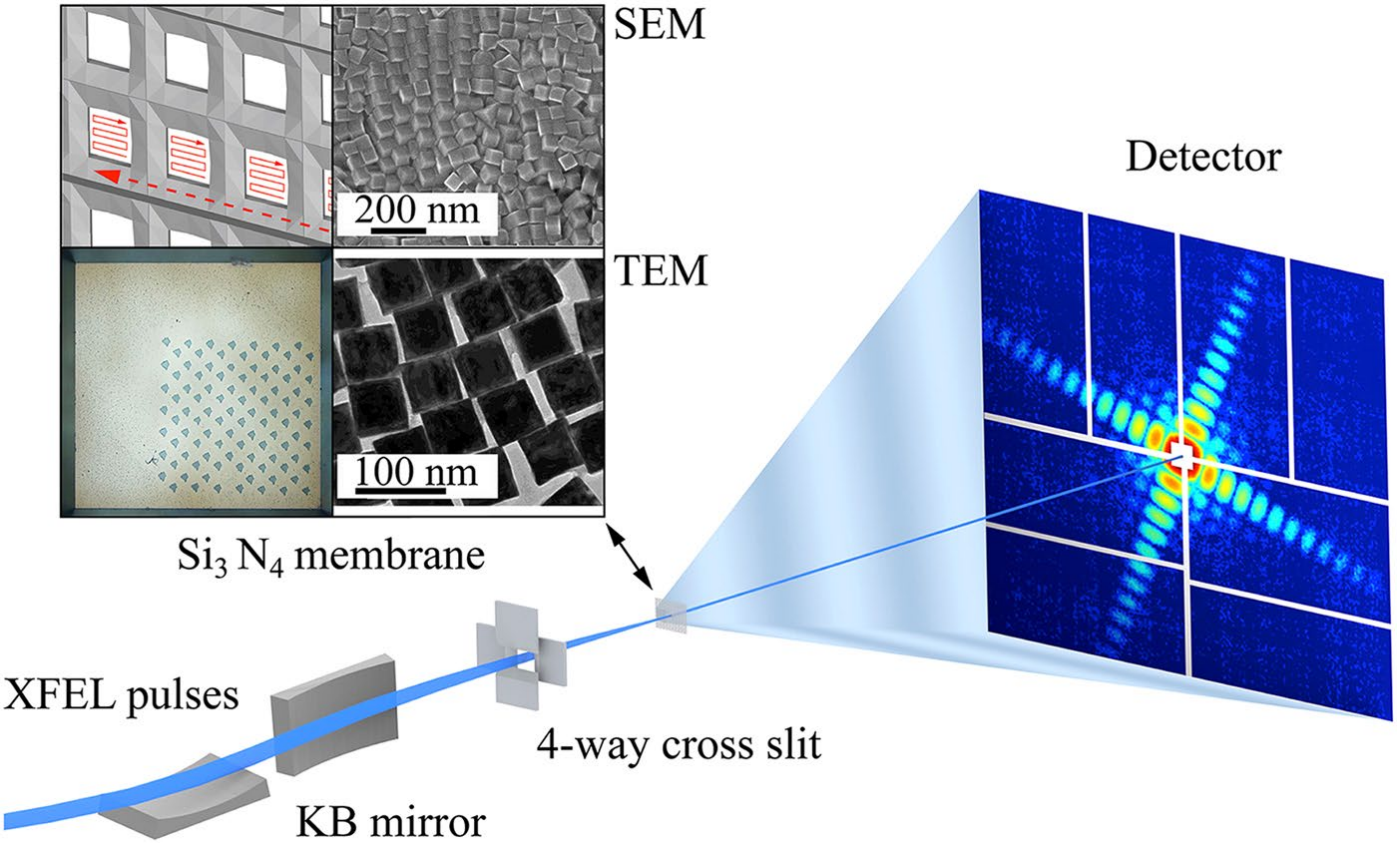
Schematic layout of the single-shot 3D diffractive imaging set-up. XFEL pulses with an energy of 6 keV and a pulse duration of 5–6 fs were focused to a 1.5 μm spot by a pair of K-B mirrors. A four-way cross slit was used to eliminate the parasitic scattering from the mirrors. Au/Pd core-shell nanoparticles with a monodisperse shape and size distribution (insets) were supported on a 100-nm-thick Si_3_N_4_ membrane grid and raster scanned relative to the focused beam. Each intense x-ray pulse produced a single-shot diffraction pattern, recorded by an octal multi-port charge-coupled device. A small hole was created on the Si_3_N_4_ membrane after a single exposure (insets).

Au/Pd core-shell nanoparticles were synthesized by a seed mediated growth method from soluble precursors^46^. First, Au nanoparticles with truncated cubic shapes were prepared as the cores. Then followed the epitaxial growth of a Pd shell on the cubic Au core followed, upon which the composite nanoparticles adopted a perfect cubic shape. Scanning electron microscope and Transmission Electron Microscope (TEM) show a monodisperse shape and size distribution of the nanoparticles (Fig. 1 insets). The formation of the Au/Pd core-shell structure was also implicated by the alternating bright and dark fringes in the TEM image caused by the superposition of two misfit crystalline lattices in a core-shell construction. The XFEL experiment was conducted using the SPring-8 Angstrom Compact Free Electron Laser^36^. Figure 1 shows the schematic layout of the single-shot 3D diffractive imaging experiment. X-ray pulses with an energy of 6 keV and a repetition rate of 10 Hz were focused to a 1.5 μm spot by a pair of Kirkpatrick-Baez (K-B) mirrors. Each pulse contained ~10^11^ photons with a pulse duration of 5–6 fs (Supplementary Fig. 1). Nanoparticles were deposited onto a 100-nm-thick Si_3_N_4_ membrane grid and inserted into a multi-application x-ray imaging chamber^47^, where the sample was scanned relative to the x-ray pulses. Single-shot x-ray diffraction patterns were measured by an octal multi-port charge-coupled device with 2,048 × 2,048 pixels and a pixel size of 50 × 50 μm^48^, placed at a distance of 1.5 m from the sample. The nanoparticles were destroyed after the impinging of x-ray pulses, leaving small holes on the Si_3_N_4_ membrane (Fig. 1 inset). A total of 39,151 diffraction patterns were acquired consisting of no hits, partial hits, single-particle hits, and multiple-particle hits. To separate single-particle hits from the other hits, we used a data screen approach developed elsewhere^43^. We first pre-defined a common region across all the diffraction patterns and calculated the average intensity within the region of each pattern. Based on the average intensity, we divided the diffraction patterns into sub-groups, consisting of no hits, partial hits, single-particle hits, and multiple-particle hits. From the sub-group of single-particle hits, 34 representative diffraction patterns were selected for further analysis.

**Figure 2:**
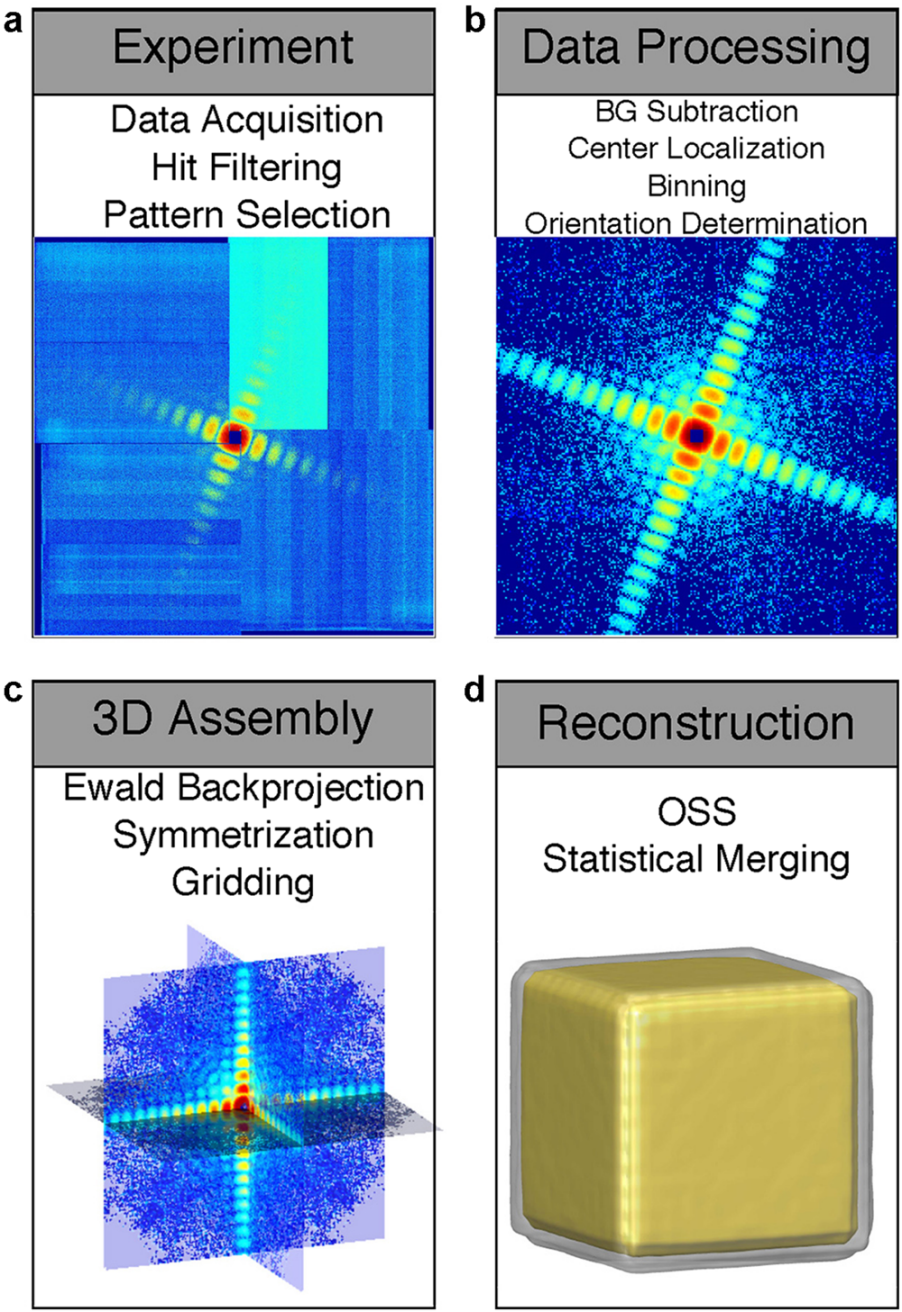
Semi-automated data analysis and 3D reconstruction pipeline. (**a**) A large number of diffraction patterns were experimentally collected consisting of no, partial, single, and multiple hits by XFEL pulses. High-quality single-hit diffraction patterns were selected from these patterns. The different colors in the pattern are due to the difference of the read-out noise of the detector segments. (**b**) After background subtraction and center localization, each diffraction pattern was binned by 9 × 9 pixels to enhance the signal-to-noise ratio and the orientation of the pattern was determined. (**c**) By taking advantage of the curvature of the Ewald sphere and symmetry intrinsic to the nanoparticle, a single-shot diffraction pattern was used to produce a 3D Cartesian grid of the Fourier magnitudes by a gridding method. (**d**) The 3D phase retrieval was performed by the OSS algorithm. Among 1,000 independent reconstructions, the top 10% with the smallest R-factors were averaged to obtain a final 3D reconstruction for each single-shot diffraction pattern.

The 34 diffraction patterns were processed and reconstructed by using a semi-automated 3D data analysis pipeline, shown in Fig. 2. From each diffraction pattern, the background was subtracted based on the most recent background exposure. An additional flat background subtraction was required, the value of which was determined by first smoothing and thresholding each pattern to determine the background region. The final subtracted value was determined by the average nonnegative pixel intensity in the background region multiplied by a single scaling factor, whose value was optimized based upon the quality of the resulting reconstructions. Due to the strong diffraction signal and large oversampling ratio, this was a sufficient background treatment for this experiment, but we note that for more low-contrast samples a more sophisticated background analysis would likely be preferable^49^. The center of each diffraction pattern was determined based on the centro-symmetry of the diffraction intensity. Since the diffraction patterns have large oversampling ratios^50^, each pattern was binned by 9 × 9 pixels to enhance the signal-to-noise ratio^51^. Supplementary Figure 2 shows the 34 processed single-shot diffraction patterns, in which the diffraction signal is limited by the size of the detector. The orientation of each single-shot diffraction pattern can in principle be determined by the self-common arc method^43^. But the 34 diffraction patterns in this experiment were oriented close to the four-fold symmetry axis as the majority of nanocubes sit flat on the surface of Si_3_N_4_ membranes (Fig. 1 insets). This allowed us to develop a simpler approach to refine the orientation of each diffraction pattern. We first estimated the size of a nanocube based on the speckle size and experimental parameters. We then slightly changed the orientation of the nanocube and calculated the corresponding diffraction pattern. By minimizing the difference between the calculated and measured diffraction patterns, we determined the orientation of each diffraction pattern with an angular precision of ~0.5°.

Each diffraction pattern was then projected onto the surface of the Ewald sphere^39^. By taking into account of the curvature of the Ewald sphere and the 48 octahedral symmetry operations, a 3D Cartesian grid of the Fourier magnitudes was assembled by the following interpolation approach,

1 $$|{F}_{obs}(\overrightarrow{k})|=\frac{{{\rm{\Sigma }}}_{i}\frac{|F({\overrightarrow{k}}_{i})|W({\overrightarrow{k}}_{i})}{\Delta {\Omega }_{i}|{\overrightarrow{k}}_{i}-\overrightarrow{k}|}}{{{\rm{\Sigma }}}_{i}\frac{W({\overrightarrow{k}}_{i})}{|{\overrightarrow{k}}_{i}-\overrightarrow{k}|}}\,;\,\,W({\overrightarrow{k}}_{i})=\{\begin{array}{c}1,\,\frac{|{\overrightarrow{k}}_{i}-\overrightarrow{k}|}{{\rm{\Delta }}p} < {d}_{c}\\ 0,\,\frac{|{\overrightarrow{k}}_{i}-\overrightarrow{k}|}{{\rm{\Delta }}p}\ge {d}_{c}\end{array}$$

where $\,|{F}_{obs}(\overrightarrow{k})|\,\,$is the interpolated Fourier magnitudes on the 3D Cartesian grid point ($\overrightarrow{k}$).$\,|F({\overrightarrow{k}}_{i})|$ is the measured Fourier magnitudes of the *i*^th^ pixel projected onto a point ${\overrightarrow{k}}_{i}$ on the surface of the Ewald sphere, $W({\overrightarrow{k}}_{i})$ represents a spherical interpolation kernel of radius ${d}_{c}$ (where ${d}_{c}$ = 0.7 voxels in this case), $\Delta {\Omega }_{i}$ is the solid angle subtended by the *i*^th^ pixel of the detector and $\Delta p$ is the pixel size in reciprocal space. When the diffraction pattern has a large oversampling ratio and the Fourier magnitudes change smoothly^50^, this interpolation approach is computationally efficient and accurate.

**Figure 3:**
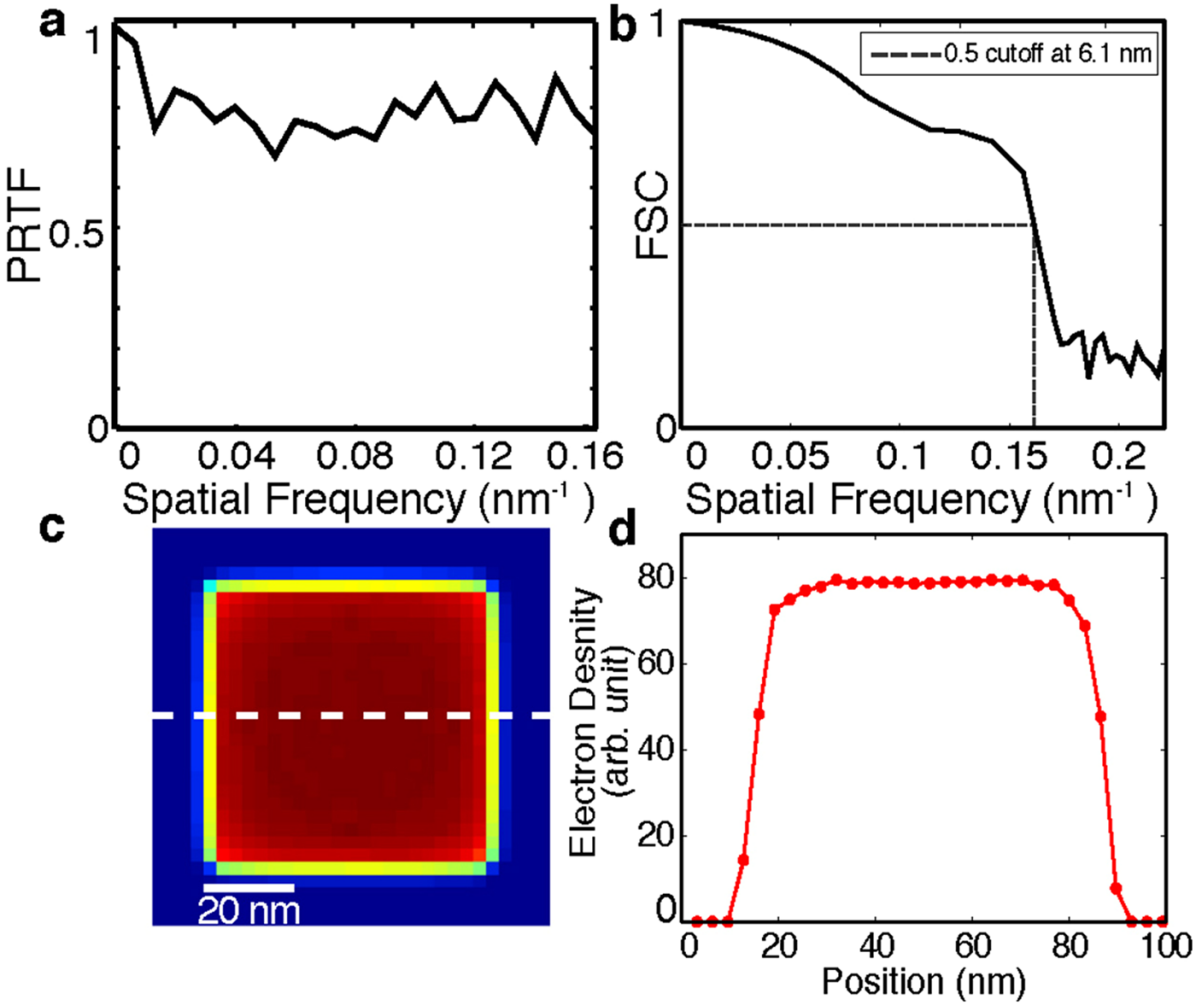
Quantitative analysis of the 3D reconstruction. (**a**) Average Phase Retrieval Transfer Function (PRTF) across all of the multiple experimental reconstructions for all 34 diffraction patterns. (**b**) Average Fourier shell correlation (FSC) between every pair of the 34 reconstructed nanoparticles, indicating a 3D resolution of 6.1 nm based on the criterion of FSC = 0.5. (**c**) Central 32-nm-thick slice of a final 3D reconstruction with an overlaid line scan plotted in (**d**), showing the electron density variation of the Au core and Pd shell.

Using Eq. (1), we produced a 3D Cartesian grid of the Fourier magnitudes for each single-shot diffraction pattern. A fraction of the grid points were filled in by the measured data and the remaining points were set as undefined. The phase retrieval was carried out by the oversampling smoothness (OSS) algorithm^52^. A total of 1,000 independent, randomly seeded 3D reconstructions were performed for each 3D grid of the Fourier magnitudes. Each reconstruction consisted of 1,000 iterations of OSS with ten progressive filters, positivity constraint and a loose cubic support with a linear oversampling ratio of approximately 9. The algorithm iterated between real and reciprocal space. The positivity and support constraints were applied in real space and the measured grid points were enforced in reciprocal space, while the undefined points were iteratively determined by the algorithm. An R-factor, defined as the sum of the difference between measured and calculated Fourier magnitudes normalized by the sum of the measured Fourier magnitudes, was used to monitor the convergence of the iterative algorithm. The phase retrieval transfer function (PRTF) was also calculated^15^ (Fig. 3a). After 1,000 iterations, the majority of 1,000 independent reconstructions had converged and the top 10% with the smallest R-factors were averaged to obtain a final 3D reconstruction. Because the quantity of data obtained during an XFEL experiment is so high, we have implemented a semi-automated pipeline for diffraction pattern selection, data analysis and 3D reconstruction (Fig. 2), allowing for visualization of the final 3D reconstructions during the experiment. By using this pipeline, we obtained the final reconstructions of the 34 single-shot diffraction patterns (Supplementary Fig. 3). Figure 2d and movie 1 show the iso-surface renderings of a representative final reconstruction, in which the core and shell structures are clearly visible. To quantify the resolution, we calculated the Fourier shell correlation (FSC) between the final reconstructions of different single-shot diffraction patterns, which has been widely used to estimate the resolution in cryo-electron microscopy^45^. Based on the criterion of FSC = 0.5, we estimated a 3D resolution of 6.1 nm was achieved for the reconstructions (Fig. 3b). The sudden drop of the FSC curve corresponds to the cut-off of the diffraction intensity by the detector edge, indicating that either the use of a larger detector or shortening the distance between the sample and the detector will improve the achievable resolution.

### Extracting structural information beyond the diffraction signal

**Figure 4:**
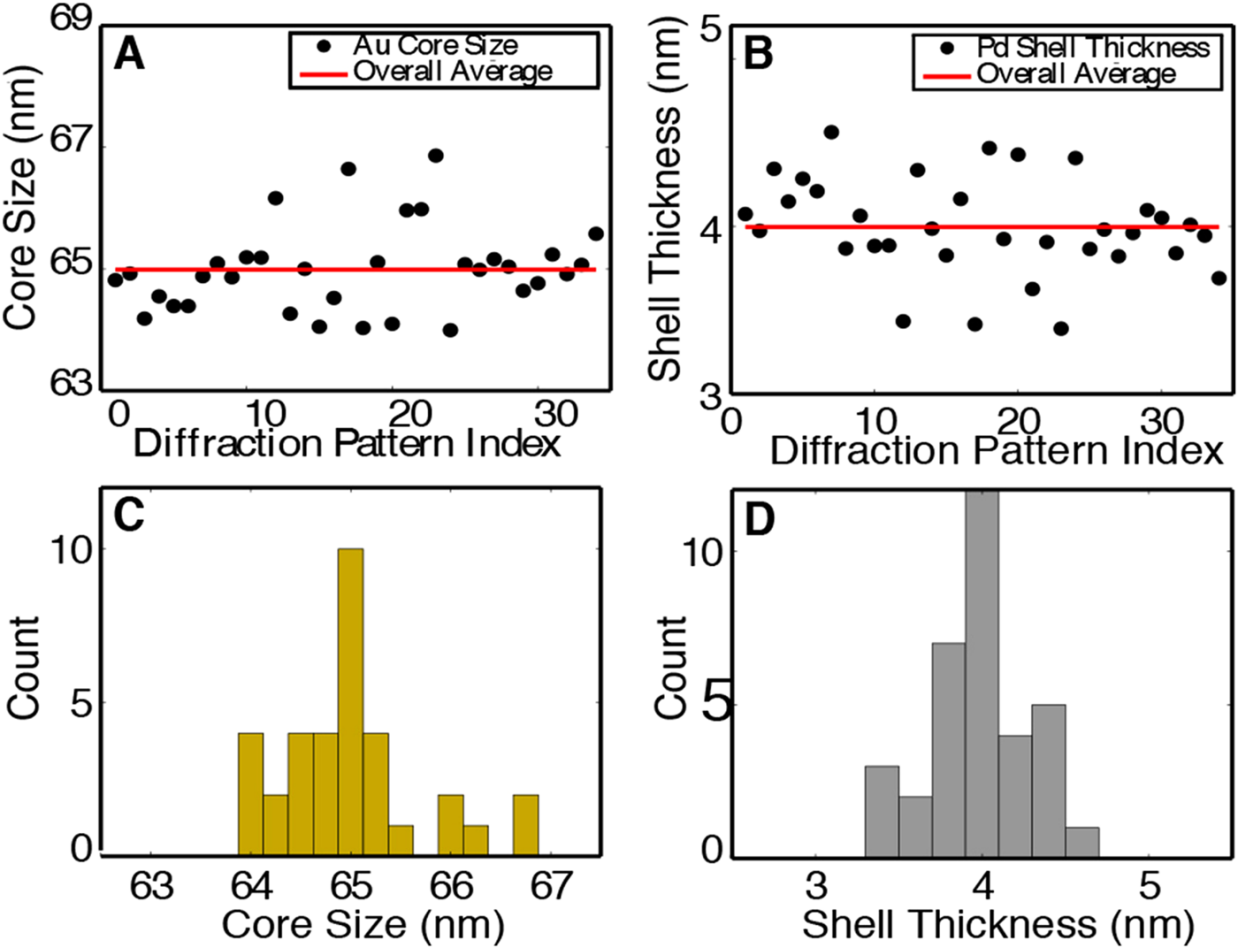
Experimental implementation of 3D super-resolution CDI of core-shell nanoparticles. (**A**) and (**B**) The distribution of the core size and shell thickness obtained from 34 single-shot diffraction patterns. Each data point shows the mean and standard deviation of the top 10% of 1,000 independent reconstructions for a single-shot diffraction pattern. The horizontal red lines indicate the average core size and shell thickness across all 34 nanoparticles. (**C**) and (**D**) The core/shell distribution of the 34 nanoparticles, indicating the Au core size and the Pd shell thickness are 65.0 ± 1.0 nm and 4.0 ± 0.5 nm, respectively, which are beyond the diffraction signal resolution (6.1 nm).

To precisely determine the size and elemental specificity of the core-shell nanoparticles, we implemented a super-resolution 3D CDI technique. This technique exploited the quantitative 3D reconstruction and used model fitting to achieve a resolution beyond the diffraction signal. Specifically, for each of the top 10% independent reconstructions resulting from a single-shot diffraction pattern, a 3D model was created at five times the voxel resolution of the reconstructed structure. The value of five times was chosen as higher values were found to produce extremely similar resulting models at the cost of much large computation times. The model was binned and compared with the reconstruction using an error metric,

2 $$Err=\frac{{\sum }_{\overrightarrow{r}}|{\rho }_{rec}(\overrightarrow{r})-{\rho }_{mod}(\overrightarrow{r})|}{{\sum }_{\overrightarrow{r}}{\rho }_{mod}(\overrightarrow{r})}$$

where ${\rho }_{rec}(\overrightarrow{r})$ and ${\rho }_{mod}(\overrightarrow{r})$ are the electron intensity of the reconstruction and model, respectively. By varying the core size, shell thickness, and ratio of core to shell density, a series of errors were computed using Eq. (2) and the model with the lowest error was recorded for the reconstruction. For all the top 10% independent reconstructions from a single-shot diffraction pattern, the parameters of the recorded models were averaged to obtain the core size, shell thickness, and ratio of core to shell density (Fig. 4). To validate this technique, we applied it to 34 single-shot diffraction patterns, each of which was measured from a different core-shell nanoparticle. Figure 4 shows the distribution of the average core size, shell thickness, and ratio of core to shell density for the 34 nanoparticles. The core size is between 64 and 67 nm, while the shell thickness is within 3–5 nm. By statistically averaging the 34 nanoparticles, we obtained a 4.0 ± 0.5 nm thick shell of Pd surrounding a uniform 65.0 ± 1.0 nm Au core, indicating that we can achieve resolution better than the diffraction signal (6.1 nm). The average intensity ratio between the Au core and Pd shell was 1.69, which agrees with the tabulated scattering factor ratio of 1.64 within a 3% error^53^.

### Simulated model-fitting

**Figure 5:**
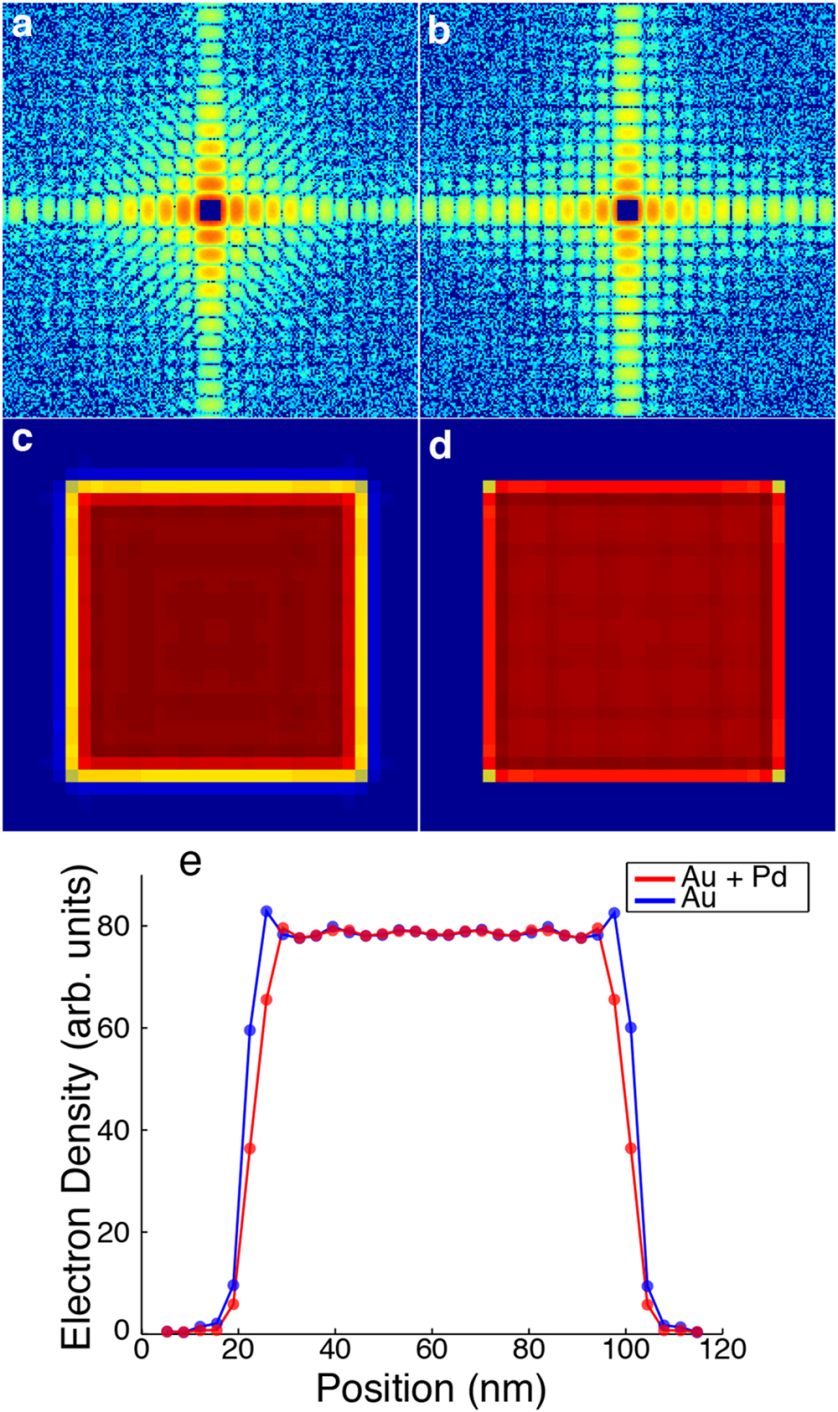
Numerical simulations on 3D super-resolution CDI of nanoparticles. Noisy diffraction patterns were calculated from a core/shell model with a 65 nm Au core and a 4 nm Pd shell (**a**) and a solid cubic model of 73 nm Au (**b**). The central data in the diffraction pattern were removed to simulate a beam stop. The top 10% of 1,000 independent reconstructions were averaged and the central 20 nm sections are shown for the Au/Pd core-shell model (**c**) and the solid Au model (**d**). The small internal density variation in the reconstruction is because i) the simulated diffraction patterns were cropped to match the maximum resolution observed experimentally and ii) symmetry was enforced in assembling a 3D grid of the Fourier magnitudes. (**e**) Line scans through the center of the corresponding reconstructions of the Au/Pd core-shell and the solid Au model.

To examine and validate the fitting procedure used, we performed a simulated analysis of the entire experiment using two different models. The first model consisted of a 65 nm Au core with a 4 nm Pd shell, and the second is a 73 nm solid Au cube with no shell. Each model was generated using a pixel size of 0.61 nm, equivalent to 1/5^th^ the observable pixel size in the experiment. Using these models, diffraction patterns were computed using 6 keV x-rays, 10^11^ photons per pulse, and a 1.5 micron spot size. The patterns were then cropped to exactly match the maximum resolution observed experimentally based on detector geometry. High Poisson noise was added to approximately the same level as that observed in the experiment and the central data were removed to simulate a beam stop (Fig. 5a,b). These diffraction patterns were each reconstructed 1,000 separate times using OSS with 1,000 iterations, 10 progressive filters, and a loose cubic support. The top 10% of reconstructions with smallest R-factors were retained for further analysis. These top reconstructions were used as input for the same model-fitting procedure described in the experiment and then averaged to produce the final reconstruction for each of the two original models.

The simulation results are shown in Fig. 5. Even at this resolution, characteristic differences in the diffraction patterns are readily observable between the core-shell and solid models (Fig. 5a,b). Blurring near the edges, which is to be expected at this resolution, is observed in both reconstructions (Fig. 5c,d); however, the intensity falloff is more pronounced in the solid model than in the core-shell one (Fig. 5e.). For the Au-Pd model, the average model-fitting result was a 65.3 nm Au core with a 3.7 nm Pd shell, and for the Au model the optimal result was a 71.3 nm Au core with a 0.67 nm Pd shell. In both cases, the overall size of the structure was determined correctly within less than 1 nm, and the small deviation between the fitted and true values of the two models is due to high Poisson noise added to the diffraction patterns. These results further validate the feasibility of using super-resolution CDI to extract 3D structural information beyond the diffraction signal.

### Conclusions and future perspectives

We demonstrated quantitative 3D imaging of Au/Pd core-shell nanoparticles with elemental specificity using XFEL pulses. These core-shell structures are representative of a vast library of nanoparticles with varying chemical, catalytic, optical and electronic properties^1–4^. We developed a semi-automated and quantitative routine for analyzing nanostructures, and applied it to 34 isolated nanoparticles. Using the curvature of the Ewald sphere and symmetry intrinsic to the nanoparticle, we reconstructed highly reproducible 3D structures from single-shot diffraction patterns with a 3D resolution of 6.1 nm. Furthermore, we implemented a 3D super-resolution CDI technique to extract structural information beyond the diffraction signal. By taking advantage of the quantitative 3D reconstruction, our super-resolution technique determined the Au core size and the Pd shell thickness to be 65.0 ± 1.0 nm and 4.0 ± 0.5 nm, respectively. The quantified electron density of the core and shell structure matches the tabulated scattering factor ratio of Au/Pd within a 3% deviation. We validated this 3D super-resolution CDI technique by using 34 independently reconstructed nanoparticles as well as through numerical simulations comparing the results of Au/Pd core-shell and solid Au models.

The implication of this work is twofold. First, although we used core-shell nanocubes as a model system to demonstrate the quantitative characterization ability, this method could in principle be applied to characterize the 3D structure of a wide range of nanoparticles with octahedral, icosahedral, cuboctahedral, decahedral, and trisoctahedral symmetry. Second, using the experimentally measured results as direct input, we constructed an epitaxial growth model for the Au/Pd core-shell nanoparticles and performed energy minimization and MD simulations (Supplementary Methods and Supplementary Fig. 5). With further improvement of the instrumentation and the peak power of the XFEL pulse, sub-nanometer CDI resolution can in principle be achieved. Thus, the combination of CDI methods and first-principles calculations such as MD could be a powerful tool to probe the structure-property relationship of nanomaterials.

### Data availability

The raw and processed 34 diffraction patterns and the source code to analyze the data are freely available at http://www.physics.ucla.edu/research/imaging/SuperResolutionCDI.

## Electronic supplementary material

Supplementary Information

Video 1
