# Supplementary material for: Single-shot 3D coherent diffractive imaging of core-shell nanoparticles with elemental specificity
Source: Sci Rep. 2018 May 29;8:8284. doi: 10.1038/s41598-018-26182-1 (PMC5974371; doi:10.1038/s41598-018-26182-1)
Supplement: Supplementary file 1 — Supplementary Information [file 41598_2018_26182_MOESM1_ESM.docx]

**Supplementary Information**

**Single-shot 3D coherent diffractive imaging of core-shell nanoparticles with elemental specificity**

Alan Pryor, Jr, Arjun Rana, Rui Xu, Jose A. Rodriguez, Yongsoo Yang, Marcus Gallagher-Jones, Huaidong Jiang, Krishan Kanhaiya, Michael Nathanson, Jaehyun Park, Sunam Kim, Sangsoo Kim, Daewoong Nam, Yu Yue, Jiadong Fan, Zhibin Sun, Bosheng Zhang, Dennis F. Gardner, Carlos Sato Baraldi Dias, Yasumasa Joti, Takaki Hatsui, Takashi Kameshima, Yuichi Inubushi, Kensuke Tono, Jim Yang Lee, Makina Yabashi, Changyong Song, Tetsuya Ishikawa, Henry C. Kapteyn, Margaret M. Murnane, Hendrik Heinz, Jianwei Miao

**Molecular dynamics simulations of the Au/Pd core-shell nanoparticle with the measured results as direct input**

The epitaxial growth model was built by first constructing a 65x65x65 nm^3^ Au core. Six regions, one for each face of the Au core, were defined in LAMMPS^54^ to create Pd atoms with an initial velocity towards the center of the nanoparticle. When the atoms collide with the nanoparticle, they become bound due to Van der Waals interactions. One atom was added to each face for every step of the simulation until the shell reached a thickness of 4 nm. Any atoms not in the core shell particle were removed, and the resulting structure was used as the starting structure. The total number of the Au and Pd atoms in the model is 16,384,000 and 6,375,018, respectively.

The initial model of 73×73×73 nm^3^ size was placed in a larger periodic simulation box (85×85×85 nm^3^). First, 1000 steps of energy minimization were performed via the conjugate gradient algorithm to relax the arbitrary spacing of model parts and gaps to reach a minimum energy structure. Subsequently, 1 ns MD was carried out in the NVT ensemble at 298.15 K, recording thermodynamic properties every 500 time-steps and snapshots every 5000 fs. A time-step of 2.5 fs and a spherical cutoff of the pairwise summation of van-der-Waals interactions at 1.2 nm were employed. A representative model in equilibrium was constructed by superposition of 50 snapshots over the last 250 ps of the simulation (Supplementary Fig. 5). The INTERFACE force field (CVFF-INTERFACE)^55-57^ and the program LAMMPS^54^ were used for all calculations. The total compute time was about 3 million core hours at ALCF, OLCF, and Summit at the University of Colorado.

**Supplementary** **Figure 1.** Intensity correlation measurements of the pulse duration of SACLA at 10 keV using an autocorrelation technique^58^. Gaussian fitting of the intensity correlation curve gave a full width of half maximum (FWHM) of 7.8 ± 0.3 fs. After considering a deconvolution factor, the pulse duration was estimated to be 7.8 fs / $\sqrt{2}$ = 5.5 fs. The autocorrelationtechnique was also applied at 6 keV, resulting in an estimated pulse duration of ~5 fs (FWHM)^59^. Based on these experimental measurements, we concluded that the pulse duration in our experiment is ~5-6 fs (FWHM).


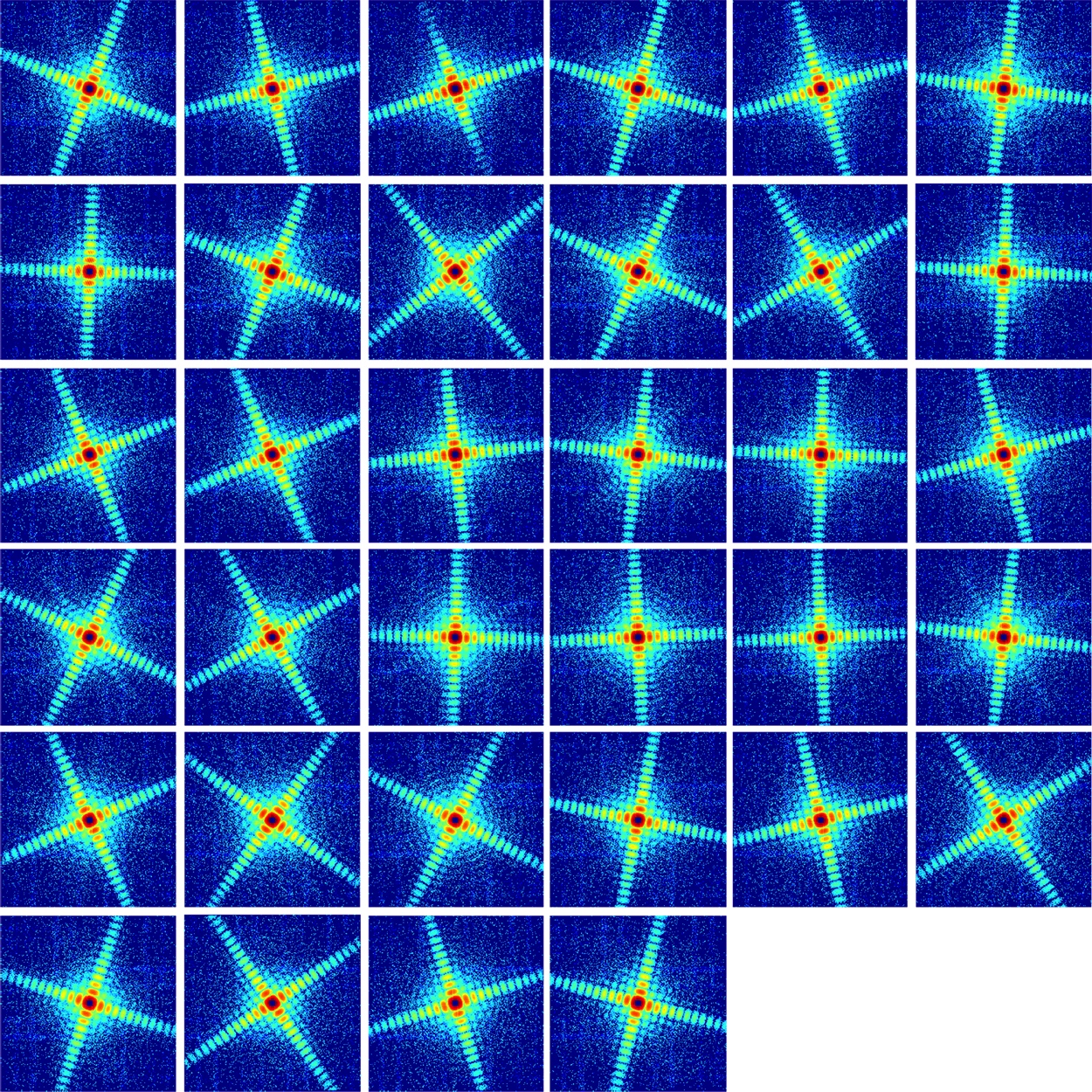


**Supplementary** **Figure 2.** 34 processed single-shot diffraction patterns, each of which was measured by impinging a very intense and short XFEL pulse on a Au/Pd core-shell nanocube. The orientation of these selected patterns is close to the four-fold symmetry axis as the majority of nanocubes sit flat on the surface of a Si_3_N_4_ membrane.


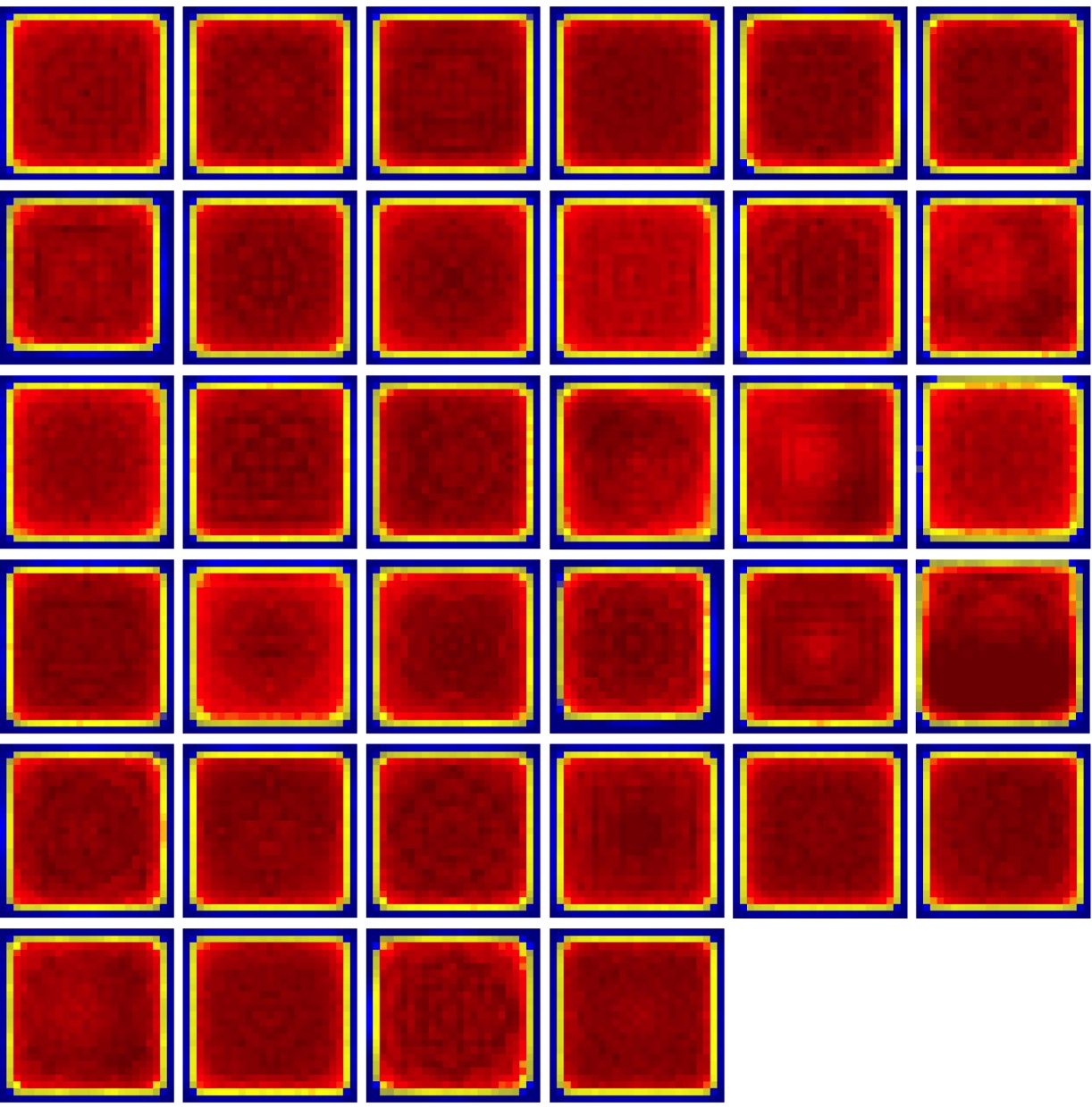


**Supplementary** **Figure 3.** Central 32-nm-thick slices of the final 3D reconstructions for the 34 single-shot diffraction patterns. The red color represents the Au core and the yellow is the Pd shell.


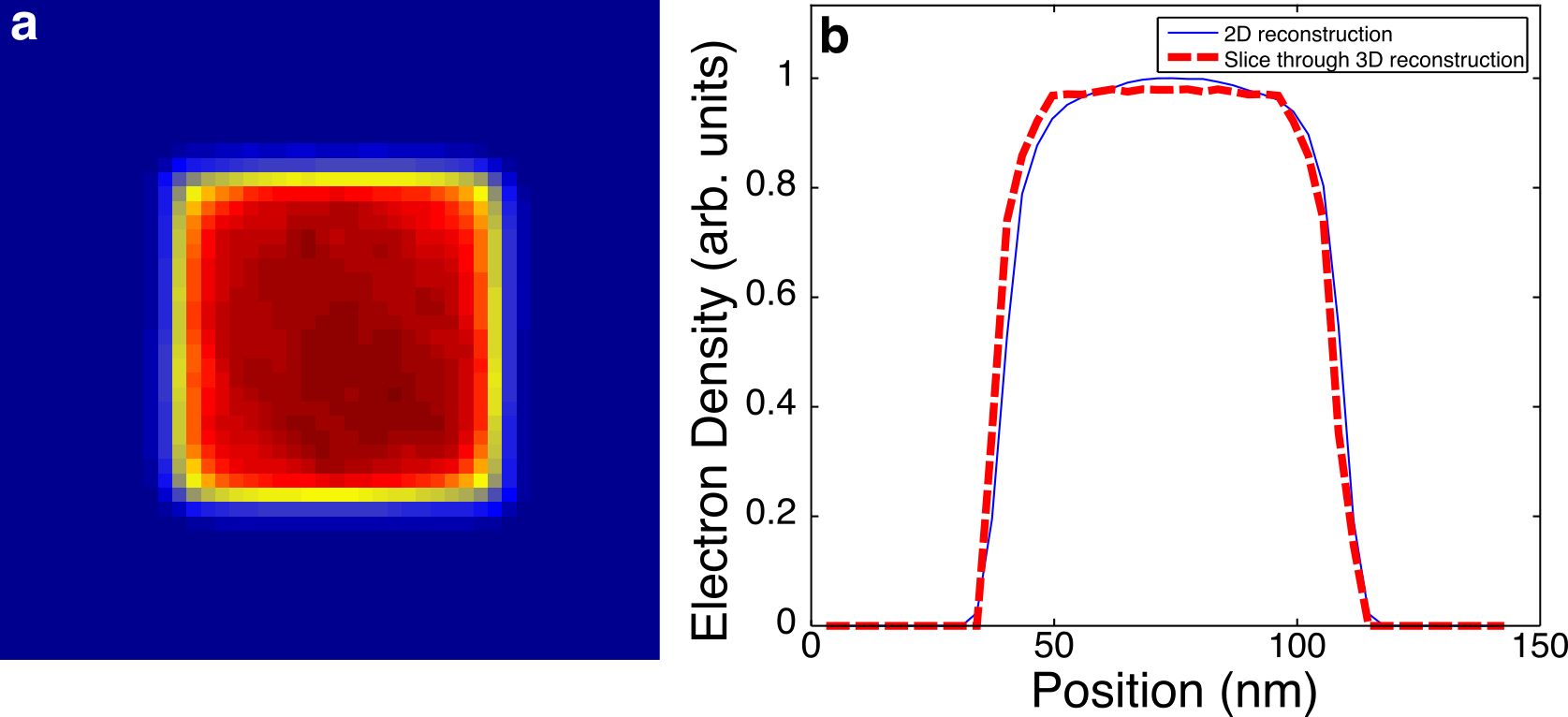


**Supplementary** **Figure 4.** Comparison of results of reconstructing an experimental diffraction pattern in 2D vs a slice through a 3D reconstruction. **a**, 2D reconstruction using one of the 34 characteristic diffraction patterns and averaging the top 100 reconstructions out of 1,000 independent OSS runs. **b.** Line scan through the central 5 pixels of the 2D reconstruction and the same line scan through a central slice of the 3D reconstruction resulting from the same diffraction pattern. The effect of limiting the reconstruction to 2D is to reconstruct a projection, which has features that are less sharp than in 3D.

**
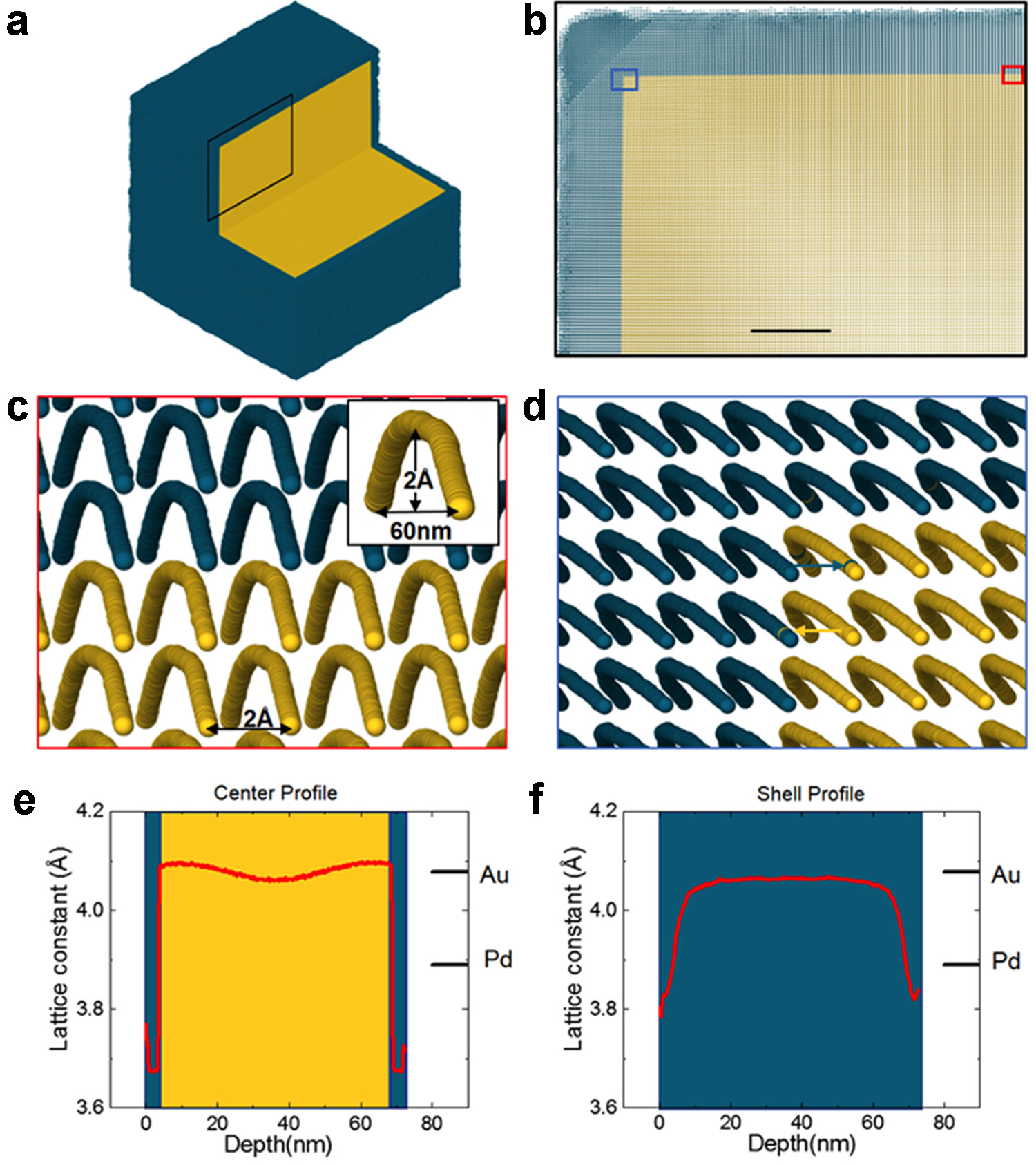
**

**Supplementary** **Figure 5.** Large-scale MD simulations of the Au/Pd core-shell nanoparticle by using experimental CDI results as direct input. **a**, An epitaxial growth model in equilibrium, consisting of a Au core of 65x65x65 nm^3^ and a Pd shell of 4 nm thick with a total of ~22.76 million atoms. **b,** Magnified view of the rectangular region in (**a**), showing the distortions at the edges and corner. Scale bar: 5 nm. **c** and **d,** Magnified views of the blue and red rectangular regions in (**b**), respectively. The arc-like atomic deformations try to force the particle towards a spherical shape at the interface. The arrows indicate diffused atoms at the Au/Pd interface. Scale bars: 0.5 nm. **e,** Lattice constants perpendicular to the Au/Pd interface through the center of the model. The two black horizontal bars indicate the Au and Pd bulk lattice constants. The yellow and blue background colors represent the Au core and Pd shell regions, respectively. **f**, Pd lattice constants in the shell region parallel to the Au/Pd interface.

**Supplementary** **Movie 1**. Iso-surface renderings of a reconstructed core-shell nanocube, showing a Au core of 65.0±1.0 nm in size surrounded by a Pd shell of 4.0±0.5 nm in thickness.

**References**

1. Plimpton, S. Fast Parallel Algorithms for Short-Range Molecular Dynamics. *Journal of Computational Physics* **117,** 1–19 (1995).
2. Heinz, H., Vaia, R. A., Farmer, B. L. & Naik, R. R. Accurate Simulation of Surfaces and Interfaces of Face-Centered Cubic Metals Using 12−6 and 9−6 Lennard-Jones Potentials. *The Journal of Physical Chemistry C* **112,** 17281–17290 (2008).
3. Heinz, H., Lin, T.-J., Kishore Mishra, R. & Emami, F. S. Thermodynamically Consistent Force Fields for the Assembly of Inorganic, Organic, and Biological Nanostructures: The INTERFACE Force Field. *Langmuir* **29,** 1754–1765 (2013).
4. Heinz, H. & Ramezani-Dakhel, H. Simulations of inorganic–bioorganic interfaces to discover new materials: insights, comparisons to experiment, challenges, and opportunities. *Chem. Soc. Rev.* **45,** 412–448 (2016).
5. Geloni, G., Kocharyan, V. & Saldin, E. Ultrafast X-ray pulse measurement method. *Report No. DESY 10-008*, arXiv:1001.3544.
6. Inoue, I. *et al.* Observation of femtosecond X-ray interactions with matter using an X-ray–X-ray pump–probe scheme. *Proc. Natl Acad. Sci. USA* **113**, 1492-1497 (2016).
